# Innate Immunity and Non-Hodgkin’s Lymphoma (NHL) Related Genes in a Nested Case-Control Study for Gastric Cancer Risk

**DOI:** 10.1371/journal.pone.0045274

**Published:** 2012-09-21

**Authors:** Sue K. Park, Jae Jeong Yang, Sohee Oh, Lisa Y. Cho, Seung Hyun Ma, Aesun Shin, Kwang-Pil Ko, Taesung Park, Keun-Young Yoo, Daehee Kang

**Affiliations:** 1 Department of Preventive Medicine, Seoul National University College of Medicine, Seoul, Korea; 2 Cancer Research Institute, Seoul National University College of Medicine, Seoul, Korea; 3 Department of Statistics, College of Natural Science, Seoul National University, Seoul, Korea; 4 National Cancer Control Research Institute, National Cancer Center, Goyang, Korea; 5 Department of Preventive Medicine, Graduate School of Medicine, Gachon University, Incheon, Korea; 6 Department of Biomedical Sciences, Seoul National University Graduate School, Seoul, Korea; University of Aberdeen, United Kingdom

## Abstract

**Objective:**

Genetic variants regulating the host immune system may contribute to the susceptibility for the development of gastric cancer. Little is known about the role of the innate immunity- and non-Hodgkin’s lymphoma (NHL)-related genes for gastric cancer risk. This nested case-control study was conducted to identify candidate genes for gastric cancer risk for future studies.

**Methods:**

In the Discovery phase, 3,072 SNPs in 203 innate immunity- and 264 NHL-related genes using the Illumine GoldenGateTM OPA Panel were analyzed in 42 matched case-control sets selected from the Korean Multi-center Cancer Cohort (KMCC). Six significant SNPs in four innate immunity (*DEFA6, DEFB1, JAK3,* and *ACAA1*) and 11 SNPs in nine NHL-related genes (*INSL3, CHMP7, BCL2L11, TNFRSF8, RAD50, CASP7, CHUK, CD79B,* and *CLDN9*) with a permutated *p*-value <0.01 were re-genotyped in the Replication phase among 386 cases and 348 controls. Odds ratios (ORs) for gastric cancer risk were estimated adjusting for age, smoking status, and *H. pylori* and CagA sero-positivity. Summarized ORs in the total study population (428 cases and 390 controls) are presented using pooled- and meta-analyses.

**Results:**

Four SNPs had no heterogeneity across the phases: in the meta-analysis, *DEFA6* rs13275170 and *DEFB1* rs2738169 had both a 1.3-fold increased odds ratio (OR) for gastric cancer (95% CIs = 1.1–1.6; and 1.1–1.5, respectively). *INSL3* rs10421916 and rs11088680 had both a 0.8-fold decreased OR for gastric cancer (95% CIs = 0.7–0.97; and 0.7–0.9, respectively).

**Conclusions:**

Our findings suggest that certain variants in the innate immunity and NHL-related genes affect the gastric cancer risk, perhaps by modulating infection-inflammation-immunity mechanisms that remain to be defined.

## Introduction

Gastric cancer is the second most common cause for cancer death in the world [1] with a marked geographic and ethnic variation in incidence and mortality [2]. *Helicobacter pylori* (*H. pylori*) infection, identified by the International Agency for Research on Cancer (IARC) as a gastric carcinogen, is a proven causal factor for gastric cancer [1], [2]. Nevertheless, some epidemiological studies have reported despite a high *H. pylori* prevalence rate, the incidence of gastric cancer is low in some Asian and African populations, known as the Asian/African enigma [3], [4]. This enigma may be due to other risk factors related to differences in individual susceptibility to gastric cancer.

In *H. pylori* infections, chronic gastric inflammation occurs and leads to an immune response that includes normal (i.e., the innate immunity) and abnormal immune responses which commonly occur in Non-Hodgkin’s lymphoma (NHL) [5]–[8]; and these processes lead to atrophic gastritis followed by metaplasia and dysplasia, and eventually connect to the development of gastric cancer [5]–[8]. Thus, it is assumed that normal immune responses triggered by *H*. *pylori* infection might be connected to abnormal immune responses in a direct and/or indirect manner and finally, might determine the final outcome such as gastric adenocarcinoma, lymphoma, and other malignancies.

Given that 1) a person’s genome is a factor for individual susceptibility; and 2) *H. pylori* infection in gastric tissue leads to inflammation that in turn induces normal/abnormal immune responses, genes coding proteins related to the host normal and/or abnormal immunity may be of importance for individual susceptibility to gastric cancer risk. Several epidemiologic studies have demonstrated that the genes related to the host immune response play a critical role in gastric carcinogenesis: 1) proinflammatory host genotypes induced hypochlorhydric/atrophic responses to gastric *H. pylori* infections, which progressing to noncardia gastric adenocarcinoma [6]; 2) a genetic variant of *TLR9,* which plays an important role in the innate immune system, was significantly associated with *H. pylori*-induced premalignant gastric changes [7]; 3) *TLR4* Asp299Gly might be a genetic susceptible factor for gastric mucosa-associated lymphoid tissue (MALT) lymphoma [8]; and 4) the *CD14* genetic variants appear to be involved in the development of gastric MALT lymphoma [9].

Based on the evidence, we hypothesized that genetic variants in the normal immune response (i.e., the innate immunity related genes) and normal immune response (i.e., the NHL related genes) could influence gastric cancer risk by modulating individual susceptibility differences. To evaluate our hypothesis, we conducted a two-stage genetic analysis including: 1) the Discovery phase to explore the 1,536 SNPs in 203 innate immunity genes and 1,536 SNPs in 264 NHL related genes using the GoldenGate (Illumina) oligonucleotide pool assay (OPA) and 2) the Replication phase that further analyzed the most significant SNPs in the Discovery phase.

## Materials and Methods

### Study Subjects

Subjects were selected from the Korean Multi-center Cancer Cohort (KMCC) in this population-based nested case-control study. The design and sampling strategy of the KMCC have been described in detail elsewhere [10]–[12]. Briefly, from 1993 to 2004, a total of 19,688 participants were recruited from four urban and rural areas in Korea. All participants 1) signed an informed consent form before entering the cohort; 2) completed detailed standardized questionnaires by personal interview; 3) donated blood and urine samples; and 4) were passively followed-up through record linkages to the national death certificate, health insurance medical records databases and the national cancer registry.

By December 2001, a total of 100 incident gastric cancer cases were newly identified according to the International Statistical Classification of Diseases and Related Health Problems 10th Revision (ICD-10, C16). Of them, 42 cases and 42 matched controls by age, sex, enrollment area, and year were included in the Discovery phase and successfully genotyped with the GoldenGate (Illumina) oligonucleotide pool assay (OPA) panels. In the Replication phase, the study populations were selected as follows: 1) according to the same methodology for case ascertainments mentioned above, 199 gastric cancer cases were additionally defined from the KMCC in December 2008 and matched 1∶1 to controls according to age (±5 years), sex, and enrollment year; 2) at the Chungnam University Hospital and Hanyang University GURI Hospital, 189 newly diagnosed gastric cancer cases were recruited from March 2002 to September 2006. They provided written informed consent and the blood samples at the time of diagnosis or prior to gastric cancer surgery; and 3) a total of 189 community-based controls matched by age (±5 years), sex and enrollment year were randomly selected from the KMCC. Of the 388 case-control matches, two cases and forty controls were eliminated due to poor genotyping rate and genomic DNA level. Finally, 386 cases and 348 controls were analyzed in the Replication phase.

Additionally, based on serology-based detection, *H. pylori* infection and CagA/VacA seropositivity were evaluated among all the study subjects included in both phases. Using the immunoblot assay Helico Blot 2.1™ (MP Biomedicals Asia Pacific, Singapore) which assures high sensitivity (99% for both *H.pylori* and CagA seropositivity) as well as specificity (98% for *H. pylori* and 90% for CagA seropositivity) among the Korean population [13], serum samples were analyzed and interrelated in accordance with the manufacturer’s instructions. Given that CagA-producing *H. pylori* are significantly associated with an increased risk of gastric cancer, *H. pylori* and CagA seropositivity were treated as potent covariates for gastric cancer and adjusted for in the statistical models.

### Ethics Statement

The study protocols of the KMCC and the genetic study were approved by the institutional review boards of Seoul National University Hospital (H-0110-084-002, and C-0603-161-170, respectively). In addition, the hospital-based gastric cancer study was approved by the Institutional Review Board of Hanyang University Hospital (IRB no.2003-4). All study participants signed an informed consent form before entering the studies.

### SNP Selection and Genotyping

In the Discovery phase, the Illumina GoldenGate™ OPA Panel was designed with 203 innate immunity and 264 NHL related candidate genes using SNPs in the SNP500Cancer project (http://snp500cancer.nci.nih.gov) with known re-sequence data and gene related carcinogenic mechanisms [14]. Focusing on a region that was 20 kb 5′ to the start of transcription (exon 1) and 10 kb 3′ to the end of the last exon (N) for each candidate gene, candidate SNPs were preliminarily screened. After this, Tag SNPs were selected from the designable set of SNPs that were part of the International HapMap Project (http://www.hapmap.org/index.html) and in later iterations, we used TagZilla (http://tagzilla.nci.nih.gov) according to the following criteria: 1) minor allele frequency (MAF) >0.05; 2) design score = 1.1; and 3) r^2^>0.8 cut-off using dbSNP from HapMap Caucasian (CEU) (data not shown). About 55% of the SNPs were located in the introns, 22% in the promoters (flanking region, UTR), 15% 3′ to the stop codon (STP), and 9% in the exons; among the SNPs that were located in exon, 73% were synonymous and 27% were nonsynonymous changes [15].

Among the 1,536 SNPs assigned to the innate immunity related genes, 384 SNPs were excluded due to a MAF <0.05 (151 SNPs), a HWE *p*-value <0.0001 (1 SNP), monomorphism (162 SNPs), and assay problems (71 SNPs). For the NHL-related panel, we removed 396 SNPs with a HWE *p*-value <0.0001 (2 SNPs), a MAF <0.05 (143 SNPs), assay problems (50 SNPs), and monomorphism (201 SNPs). Finally, the statistical analysis included 1,151 SNPs in the innate immune panel and 1,140 SNPs in the NHL panel for 42 case-control sets matched by age, sex, area, and enrollment year.

Genotyping was performed using genomic DNA extracted from peripheral blood with the Gentra Puregene Blood Kit (Gentra, Minneapolis, USA) at the Core Genotyping Facility (CGF) of the Division of Cancer Epidemiology and Genetics, National Cancer Institute. In this study, genotype completion on genomic DNA samples exceeded 95%.

For the Replication phase, six SNPs of four genes in the innate immunity gene panel (*DEFA6* rs2738120, *DEFB1* rs2702829, *DEFA6* rs13275170, *JAK3* rs2286662, *DEFB1* rs2738169, and *ACAA1* rs2239621) and 11 SNPs of nine genes in the NHL panel (*INSL3* rs10421916, *CHMP7* rs7463256, *BCL2L11* rs686952, *TNFRSF8* rs755398, *INSL3* rs11086080, *RAD50* rs17772583, *CASP7* rs11196422, *CHUK* rs2230804, *CD79B* rs2003549, *CLDN9* rs11862306, and *TNFRSF8* rs641941) were selected in accordance with the following criteria: the SNPs with 1) a permutated *p*-value<0.01; and/or 2) Benjamini-Hochberg false discovery rate (BH-FDR) corrected *p*-values <0.2 on the most significant SNPs of all the genes in the Discovery phase. Genotyping was performed using the Illumina VeraCode GoldenGate Assay with BeadXpress according to the manufacturer’s instructions (Illumina, San Diego, CA, USA) [16]. To ensure the reliability of the genotyping methods in the two phases, 188 samples were genotyped twice by each method. The concordance rate was >98.4%. Two cases and forty controls with insufficient DNA or a genotyping call rate <90% were excluded.

### Statistical Analysis

Hardy-Weinberg equilibrium (HWE) in the control group was evaluated using the chi-square test or Fisher’s exact test with a cut-off level of HWE <0.0001. Selected characteristics were evaluated using χ^2^ for categorical variables and *t* tests for continuous variables. We evaluated the association between individual SNPs and gastric cancer risk based on both the raw and permutated *p*-values that was computed using the Likelihood ratio test (LRT) with 1 degree of freedom in the trend (additive) model. The trend test assumes a dose response effect (i.e., linear effect) with an increasing number of variant alleles. Permutated *p*-values were estimated by 10,000 permutation tests. Odds ratios (ORs) and 95% confidence intervals (95% CIs) were estimated using logistic regression model adjusting for age, cigarette smoking status (yes *vs.* no), *H. pylori* infectivity, and CagA virulent factors (positive *vs.* negative) which are alleged risk factors for gastric cancer although the variables were not significant in our data. When data are sparse and asymptotic approximations based on logistic regression models are unreliable, exact inference should be considered [17]. Thus, if the genotype frequency in either the cases or controls was less than 10% (N = 4), exact logistic regression analysis was conducted to calculate the ORs and 95% CIs.

To avoid spurious associations with false positive outcomes, we selected all SNPs with the most significant *p*-value for each gene, and the Benjamini-Hochberg false discovery rate (BH-FDR) corrected *p*-values of each SNP was computed according to the number of genes [18]. We primarily selected significant SNPs with a permutated *p*-value<0.01 and a BH-FDR *p*-value<0.2.

In the Replication phase, the most significant SNPs identified in the Discovery phase were re-evaluated. Based on the additive or recessive models, gastric cancer risk was estimated as ORs and 95% CIs using logistic regression model adjusting for the same covariates mentioned above. To summarize the results from the Discovery and the Replication phases, pooled- and meta-analyses were conducted. Using the fixed effect model, the summarized ORs and 95% CIs were computed. In addition, heterogeneity across the studies was evaluated by the Cochran Q statistics [19].

All statistical analysis was conducted with the PLINK software version 1.06 (http://pngu.mgh.harvard.edu/purcell/plink) and SAS software version 9.1 (SAS Institute, Cary, North Carolina).

## Results

Cases and controls were comparable in age, *H. pylori* infection, CagA/VacA seropositivity, drinking status, and gastric ulcer history. A marginally significant and significant different distribution for cigarette smoking between cases and controls was observed in the Replication and Pooled analyses (Table 1).

**Table 1:** General characteristics of gastric cancer cases and controls selected for the screening of 203 innate immunity- and 264 NHL-related candidate genes in the discovery, replication and pooled analyses.

		Discovery	Replication	Pooled
	Unit	Cases/Controls	Cases/Controls	Cases/Controls
**Total**	Number	42/42	386/348	428/390
**Age**	Mean	63.0/62.9	61.5/63.1	62.0/63.0
**Sex**	Male, N (%)	30 (71.4)/27 (64.3)	256 (66.3)/238 (68.4)	286 (66.8)/265 (67.9)
***H.pylori*** ** infection**	Positive, N (%)	35 (83.3)/38 (90.5)	342 (88.6)/299 (85.9)	377 (88.1)/337 (86.4)
**CagA**	Positive, N (%)	36 (85.7)/39 (92.9)	355 (92.0)/308 (88.5)	391 (91.4)/347 (89.0)
**VacA**	Positive, N (%)	27 (64.3)/25 (59.5)	271 (70.2)/233 (67.0)	298 (69.6)/258 (66.2)
**Smoking status**	Smoker, N (%)	30 (71.4)/24 (57.1)	238 (61.7)/191 (54.9)**	268 (62.6)/215 (55.1)*
**Drink status**	Drinker, N (%)	20 (47.6)/23 (54.8)	239 (61.9)/206 (59.2)	259 (60.5)/229 (58.7)
**Histological type** a	Non-cardia, N (%)	22 (52.4)/N.A.	262 (67.9)/N.A.	284 (66.4)/N.A.

* Statistically significant or **marginally significant in the distribution of cases and controls.

a Defined according to the ICD-10 code: Cardia C16.0; Non-cardia C16.1–C16.6; Overlapping C16.8; and Unspecified C16.9.

Genotype frequencies for all SNPs did not deviate from the Hardy-Weinberg equilibrium (HWE) (*p*>0.0001).

In the Discovery phase, Six SNPs in four genes (*DEFA6, DEFB1, JAK3*, and *ACAA1*) among 1,152 SNPs selected from the innate immunity related genes and 11 SNPs in nine genes (*INSL3, CHMP7, CASP7, RAD50, CHUK, TNFRSF8, CD79B, BCL2L11,* and *CLDN9*) among the 1,104 SNPs in 246 NHL-related genes were significantly associated with gastric cancer risk according to the permutated *p*-values (*p*<0.01). After correcting for multiple comparisons, only two SNPs, *DEFA6* rs2738120 and *DEFB1* rs2702829, remained marginally significant for gastric cancer (FDR = 0.08 and FDR = 0.08, respectively), which showed an increased risk for gastric cancer (OR [95% CI] = 4.1 [1.6–10.4] and 2.9 [1.3–6.1], respectively). Of the NHL-related gene panel, one SNP in nine genes remained marginally significant (FDR-BH *p*-value = 0.09); *INSL3* rs10421916 was associated with a significantly decreased for gastric cancer (OR [95% CI] = 0.6 [0.3–0.97]) (Table 2).

**Table 2:** The results in the Discovery phase: Innate immunity and NHL-related gene variants and risk of gastric cancer in the Illumina OPA.

Gene	db SNP ID	Polymorphism	MAF^a^ (%)	CHR^b^	Chromosomeposition	Region	FDR-BH*p*-value c	OR (95% CI) d
***Innate immunity-related genes***								
*DEFA6*	rs2738120	C/G	C (13.9)	8	6770046	Intron	0.08	4.1 (1.6–10.4)
*DEFB1*	rs2702829	T/C	G (31.1)	8	6714615	3′STP	0.08	2.9 (1.3–6.1)
*DEFA6*	rs13275170	G/A	C (16.7)	8	6767654	3′STP	0.20	3.1 (1.3–7.6)
*JAK3*	rs2286662	A/G	T (40.5)	19	17793138	Exon	0.20	0.3 (0.1–0.8)
*DEFB1*	rs2738169	C/T	G (47.3)	8	6729968	UTR	0.20	2.1 (1.1–4.4)
*ACAA1*	rs2239621	G/A	C (48.6)	3	38148737	Intron	0.20	3.1 (1.3–7.3)
***NHL-related genes***								
*INSL3*	rs10421916	T/C	A (29.5)	19	17789987	Intron	0.09	0.6 (0.3–0.97)
*CHMP7*	rs7463256	T/C	C (48.2)	8	23157565	Intron	0.12	2.9 (1.3–6.3)
*BCL2L11*	rs686952	A/C	A (11.5)	2	111635817	Intron	0.12	4.8 (1.4–17.6)
*TNFRSF8*	rs755398	G/C	C (30.7)	1	12124682	Intron	0.12	3.4 (1.5–8.1)
*INSL3*	rs11086080	G/A	T (35.5)	19	17788041	3′STP	0.13	0.5 (0.3–0.97)
*RAD50*	rs17772583	A/G	G (46.4)	5	131981409	Intron	0.13	3.1 (1.3–7.5)
*CASP7*	rs11196422	G/A	A (20.5)	10	115433080	Intron	0.13	0.2 (0.1–0.6)
*CHUK*	rs2230804	G/A	T (48.8)	10	101967873	Exon	0.15	3.0 (1.4–6.5)
*CD79B*	rs2003549	G/A	T (7.4)	17	59362169	Intron	0.15	9.0 (1.1–79.3)
*CLDN9*	rs11862306	C/T	T (47)	16	3001929	UTR	0.16	2.6 (1.2–5.7)
*TNFRSF8*	rs641941	G/A	G (30.1)	1	12129239	3′STP	0.15	3.1 (1.3–7.0)

a Minor allele frequency among controls.

b Chromosome number.

c Adjusted FDR-BH *p*-values calculated by the most significant SNPs of all genes in analysis; *p* value of cut-off level ≤0.2.

d Adjusted for age, smoking status (never vs. ever), history of *Helicobacter Pylori* infection and CagA seropositivity.

In the combined analysis (i.e., pooled and meta analyses) that included the Discovery and Replication phases, two SNPs, *DEFA6* rs13275170 and *DEFA1* rs2738169 in the innate immunity genes and *INSL3* rs10421916 and rs11086080 in the NHL-related genes remained significantly associated with risk for gastric cancer (meta OR = 1.3 [95% CI: 1.1–1.6]; meta OR = 1.3 [95% CI: 1.1–1.5]; meta OR = 0.8 [95% CI: 0.7–0.97]; meta OR = 0.8 [95% CI: 0.7–0.9], respectively). There was no heterogeneity across the analyses (Cochran Q test, *p*>0.05) (Table 3).

**Table 3:** The results of the pooled- and meta-analyses including the discovery and replication phases: Association of most significant SNPs in the innate immunity and NHL-related gene variants and gastric cancer risk.

Gene	db SNP ID	Minorallele	*p-value*heterogeneity a	OR (95% CI)^b^Pooled-analysis	OR (95% CI)^b^Meta-analysis
**Innate immunity-related genes**					
*DEFA6*	rs2738120	C	0.02	1.3 (1.0–1.6)	1.4 (1.1–1.7)
*DEFB1*	rs2702829	G	0.04	1.3 (1.0–1.5)	1.3 (1.1–1.6)
***DEFA6***	**rs13275170**	**C**	**0.07**	**1.2 (1.0–1.5)**	**1.3 (1.1–1.6)**
*JAK3*	rs2286662	T	0.03	0.8 (0.7–1.1)	0.8 (0.6–1.l)
***DEFB1***	**rs2738169**	**G**	**0.12**	**1.2 (1.01–1.5)**	**1.3 (1.1–1.5)**
*ACAA1*	rs2239621	C	0.01	1.1 (0.8–1.3)	1.1 (0.9–1.3)
**NHL-related genes**					
***INSL3***	**rs10421916**	**A**	**0.15**	**0.8 (0.7–0.99)**	**0.8 (0.7–0.97)**
*CHMP7*	rs7463256	T	0.02	1.1 (0.9–1.3)	1.2 (0.9–1.4)
*BCL2L11*	rs686952	A	0.02	0.9 (0.7–1.4)	1.0 (0.8–1.5)
*TNFRSF8*	rs755398	G	0.01	1.1 (0.8–1.3)	1.1 (0.9–1.4)
***INSL3***	**rs11086080**	**T**	**0.19**	**0.8 (0.7–0.98)**	**0.8 (0.7–0.9)**
*RAD50*	rs17772583	G	0.02	1.1 (0.9–1.3)	1.1 (0.9–1.4)
*CASP7*	rs11196422	A	0.01	0.9 (0.7–1.2)	0.8 (0.6–1.1)
*CHUK*	rs2230804	T	0.02	1.2 (0.9–1.4)	1.2 (1.0–1.5)
*CD79B*	rs2003549	T	0.10	1.4 (0.9–2.4)	1.5 (1.0–2.5)
*CLDN9*	rs11862306	T	0.02	1.0 (0.8–1.2)	1.1 (0.9–1.3)
*TNFRSF8*	rs641941	G	0.03	1.1 (0.9–1.4)	1.2 (0.9–1.5)

a Cochran’s *p*-value of heterogeneity between the ORs (95% CIs) of discovery and those of the replication phase.

b All ORs were adjusted for age, smoking status (never *vs.* ever), *H. pylori* infection (positive *vs.* negative) and CagA seropositivity (positive *vs.* negative).

## Discussion

A two-stage genetic association study with 1,536 SNPs in 203 innate immunity related (i.e., the normal immune response related) genes and 1,536 SNPs in 264 NHL-related (i.e., the abnormal immune response related) genes was conducted to identify variants in innate immunity and NHL-related candidate genes associated with gastric cancer. In the Discovery phase, six SNPs in four innate immunity genes (*DEFA6, DEFB1, JAK3*, and *ACAA1*) and 11 SNPs in nine NHL-related genes (*INSL3, CHMP7, CASP7, RAD50, CHUK, TNFRSF8, CD79B, BCL2L11,* and *CLDN9*) were significantly associated with gastric cancer risk (permutated *p*<0.01); and after the BH-FDR test for the genes in 42 sets of cases and controls, *DEFA6* and *DEFB1* in the innate immunity gene panel and *INSL3* in the NHL-related gene panel were significantly associated with gastric cancer risk (BH-FDR *p*<0.1). In the Replication genotyping phase with more case-control sets and in the meta- and pooled analyses, *DEFA6, DEFB1*, and *INSL3* were still significantly associated with gastric cancer risk without heterogeneity across the phases.

*DEFA6* (defensin alpha 6, Paneth cell-specific) and *DEFB1* (defensin beta 1), which belong to the defensin family members, consists of 6 human beta-defensins (hBD1–hBD6) and two human alpha-defensins (hD5–hD6) and play a crucial role in the host defense mechanism, especially in relation to antimicrobial activity and cytotoxicity of neutrophils [20], [21]. Defensins inducible by inflammation or infections can resist bacterial colonization at epithelial surfaces including gastrointestinal epithelium [22]–[24]. In particular, defensin binds lipopolysaccharide on the *H. pylori* cell wall and the defensin-*H. pylori* complex activates the NF-kB signal transduction pathway for gastric carcinogenesis [25]. Recent studies indicated defensins involved in cancer development were expressed in various human tumors including gastric cancer [26]–[28]. As one of the potential agents for gastric carcinogenesis, defensins may interact with *H. pylori* infection for both resistance and protection, and their abnormal activities can induce the first step toward gastric cancer development. Thus, it is possible that some variants in *DEFA6* and *DEFB1* affect the susceptibility to gastric cancer by modifying the inflammatory response through changes in the expression or function of those proteins. Our findings suggest that *DEFA6* rs2738120 (located in the intron) and *DEFB1* rs2702829 (located in the 3′ of STP) have potential genetic effects on the development of gastric cancer. Despite the heterogeneity of the discovery and replication phase, the other locations of *DEFA6* (rs2738120) and *DEFB1* (rs2702829) were also significant in our results. Further studies should be conducted to explore the exact biological functions of the SNPs related to the defensin activity and/or cellular capacity.

The *INSL3* gene encodes the protein INSL3, which is an insulin-like hormone. The INSL3 protein is produced mainly in gonadal tissues in the human body; however, it has been identified in tumor tissues of the gastrointestinal tract [29]. Little is known about how *INSL3* gene interferes in human carcinogenesis. In addition, it is not well known whether the genetic variants of the *INSL3* gene are linked to gastric carcinogenesis, but the results of this study indicate that at least two SNPs (rs10421916 located in the intron and rs11086080 located in the 3′ of STP) in strong linkage disequilibrium (D′ = 0.86, LOD = 13.42, and r^2^ = 0.56) appear to have a strong association with a reduced risk for gastric cancer. Although experimental evidence indicating a functional effect of the SNPs on a molecular level is insufficient to date, it is speculated that *INSL3* rs10421916 and rs11086080 may affect individual susceptibility in gastric carcinogenesis. Further research including in-depth *in vivo* and animal studies should be done to clarify the causal mechanisms and the *INSL3* genetic effects involved in human gastric carcinogenesis.

A number of genes (i.e., *JAK3, CHMP7, CASP7, RAD50, CHUK, TNFRSF8, CD79B, BCL2L11*, and *CLDN9*) showed a significant association with gastric cancer in the Discovery phase, although not significant in the BH-FDR tests. *JAK3*, *TNFRSF8*, and *CHUK* might affect chronic inflammation and immunity with *H. pylori* colonization toward gastric cancer, which is important in gastric carcinogenesis as follows: 1) *JAK3* is involved in cytokine receptor-mediated intracellular signal transduction, and the cytokine receptor was commonly found in human gastric cancer tissue [30]; 2) *TNFRSF8* mediated signal transduction leads to activation of NF-κB [31]; and 3) *CHUK* is involved in NF-κB signaling pathway activated by *H. pylori* in gastric cancer cells [32]. Given that the human gastric pathogen *H. pylori* exerts much of its pathogenicity by inducing DNA damage and apoptosis in host gastric epithelial cells [33], *BCL2L11, CASP7*, and *CD79B* reported to be linked to apoptosis [34]–[38] and *RAD50* and *ACAA1* reported to be linked to the DNA damage and repair system in relation to gastric cancer development [39], [40] also showed a biological plausibility. *CLDN9* acts as a tumor suppressor gene implicated in intestinal-type gastric cancer [41].

Although this study is a two-stage genetic association study, there are several limitations to the study. First, most genes did not overcome the heterogeneity across the Discovery and Replication phases. The heterogeneity is attributed to the different statistical power in each phase: A small sample size in the Discovery phase resulted in biased ORs (i.e., over-estimated), and the results were statistically distinguished from the normal-estimated values in the Replication phase. Although the distribution of the environmental factors between the cases ascertained from a community-based cohort study and hospital-based cases was slightly different from each other, the MAFs of most SNPs had little difference. Second, stratified analyses concerning the diverse characteristics of gastric cancer (i.e., histological subtypes, cardiac *vs.* non-cardiac; diffuse type *vs.* intestinal type; and TNM stage) and/or independent risk factors (i.e., age, sex, and *H. pylori* infection status) could not be conducted because of 1) the homogeneous nature of the study population (i.e., most patients showed non-cardiac types or unspecified types, >95%; and the intestinal and diffuse types were less than 3%); and 2) a lack of detail information on the histopathology data due to passive follow-up using data linkage methods. Third, since tag SNPs were selected based on data from a Caucasian population, the Asian-specific genetic markers might be disregarded in the design stage. Finally, we could not confirm a functional effect of the SNP at a molecular level. Thus, the results should be interpreted with caution. In spite of the limitations, our study is stable against several biases that are common in retrospective designs due to its population-based nested case-control design.

In conclusion, our findings suggest *DEFA6*, *DEFB1*, and *INSL3* genetic variants are susceptibility factors for the development of gastric cancer. Further studies with a greater number of cases and a more substantial genomic coverage will allow us to elucidate the pathological mechanisms of gastric cancer. Moreover, further biological studies focused on these genes should clarify their roles in gastric carcinogenesis.

## Supporting Information

Supporting Information S1 Detailed information on the selected SNPs in 203 innate immunity genes and 264 NHL related genes: In the discovery phase.
(XLSX)
